# Sublingual Immunization with Chimeric C1q/CD40 Ligand/HIV Virus-like Particles Induces Strong Mucosal Immune Responses against HIV

**DOI:** 10.3390/vaccines9111236

**Published:** 2021-10-23

**Authors:** Dongliang Liu, Sheng Zhang, Ethan Poteet, Christian Marin-Muller, Changyi Chen, Qizhi Yao

**Affiliations:** 1 Michael E. DeBakey Department of Surgery, Division of Surgical Research, Baylor College of Medicine, Houston, TX 77030, USA; Dongliang.Liu@bcm.edu (D.L.); sheng.zhang@bcm.edu (S.Z.); ethan.poteet@bcm.edu (E.P.); marinmul@bcm.edu (C.M.-M.); jchen@bcm.edu (C.C.); 2 Center for Translational Research on Inflammatory Diseases (CTRID), Michael E. DeBakey VA Medical Center, Houston, TX 77030, USA

**Keywords:** HIV, virus-like particles, vaccine, mucosal immunization, immune response, IgA

## Abstract

Development of a vaccine that can elicit robust HIV specific antibody responses in the mucosal compartments is desired for effective prevention of HIV via sexual transmission. However, the current mucosal vaccines have either poor immunogenicity when administered orally or invite safety concerns when administered intranasally. Sublingual immunization has received more attention in recent years based on its efficiency in inducing systemic and mucosal immune responses in both mucosal and extra-mucosal tissues. To facilitate the transport of the immunogen across the sub-mucosal epithelial barrier, we found that CD91, the receptor of C1q, is prevalently expressed in the sublingual mucosal lining, and thus, a modified chimeric C1q surface conjugated CD40L/HIV VLP was generated. The ability of this chimeric C1q/CD40L/HIV VLP to bind, cross the epithelial layer, access and activate the sub-mucosal layer dendritic cells (DCs), and ultimately induce enhanced mucosal and systemic immune responses against HIV is evaluated in this study. We found that C1q/CD40L/HIV VLPs have enhanced binding, increased transport across the epithelial layer, and upregulate DC activation markers as compared to CD40L/HIV VLPs alone. Mice immunized with C1q/CD40L/HIV VLPs by sublingual administration showed higher levels of IgA salivary antibodies against both HIV Gag and Env than mice immunized with CD40L/HIV VLPs. Moreover, sublingual immunization with C1q/CD40L/HIV VLPs induced more Env- and Gag-specific IFN-γ producing T cells than the CD40L/HIV VLPs group. Interestingly, C1q/CD40L/HIV VLP immunization can also induce more mucosal homing T cells than that in CD40L/HIV VLP group. Our data suggest that incorporation of C1q to CD40L/HIV VLPs is a promising novel strategy and that the sublingual immunization can be a favorite immunization route for HIV mucosal vaccines.

## 1. Introduction

Gastrointestinal and reproductive mucosal tissues are primary sites for HIV transmission and the draining lymph nodes are susceptible to HIV infection and viral replication which can result in systemic CD4^+^ T cell deficiency [1,2]. It is believed that the generation of an HIV specific antibody and cytotoxic T-lymphocyte (CTL) response in the mucosal compartment may preserve CD4^+^ T-cell populations and provide protection against viral replication, viremia, and/or result in lower viral loads in HIV infected individuals [3]. Therefore, effective mucosal vaccines that can elicit strong mucosal, humoral, and cellular immune responses are desired for prevention of HIV infection.

Although intranasal vaccination has been reported to induce a strong mucosal immune response against exogenous pathogens, the risk of redirecting vaccine antigens to the central nervous system has raised a safety concern [4]. Sublingual immunization has received more attention in recent years based on its efficiency of inducing systemic and mucosal immune responses; including humoral and CTL responses in both mucosal and extra-mucosal tissues [5,6,7,8,9,10,11,12,13]. In HIV vaccine development, sublingual vaccination with an HIV subunit vaccine can induce strong mucosal immune responses evidenced by high levels of antibodies and CTLs detected in the mouse female genital tract [6]. By using adenoviral vector encoding HIV-Gag or HIV-envelope gp120 (Env), sublingual immunization can elicit both mucosal and systemic immunity against HIV [7,8]. These data indicate the sublingual immunization route is a promising route for mucosal vaccine development.

Studies have found that antigen size and repetitive structure are critical factors for efficient antigen presentation to B cells, and that the particulate antigens such as virus-like particles (VLPs) are better antigens than soluble proteins [14,15]. VLPs have therefore shown dramatic effectiveness as candidate vaccines [14,16,17,18]. We have previously produced a chimeric CD40L/SHIV VLPs (simian-human immunodeficiency virus-like particles) using a baculovirus expression system which contains SIV Gag, HIV gp160 envelope (cleaved to gp41 and gp120), and CD40 ligand (CD40L) by taking advantage of existing knowledge about CD40/CD40L interactions between T cells and DCs which are critical for the induction and regulation of immune responses. We showed that chimeric CD40L/SHIV VLPs were able to induce enhanced immune responses in mice both by targeting HIV antigens to DCs and also by activating DCs for T cell activation. Enhanced mucosal humoral and cellular immune responses can be achieved by intranasal immunization in the mouse model by the chimeric CD40L/SHIV VLPs [19]. Therefore, the mucosal immunogenicity of particulate sized immunogen such as VLPs should be explored in sublingual immunization route.

In order to enhance the delivery of antigen to the oral-sub-mucosal and encounter DCs, we hypothesized that modified VLPs that specifically target the oral mucosal epithelium will facilitate antigen delivery and therefore enhance antigen presentation and the adaptive immune response. C1q is the first subcomponent of the C1 complex involved in the classical pathway of complement activation and was recently reported to be the ligand of CD91, a multifunctional scavenger and signaling receptor [20]. Interestingly, CD91 is reported to be highly expressed by human salivary gland epithelial cells, which makes it a potential specific target for an oral mucosal vaccine [21].

The sub-mucosal layer of the sublingual mucosa contains a dense network of DCs, and DCs rely on a number of specialized receptors to facilitate the uptake and intracellular accumulation of antigens. Studies have demonstrated that the targeted delivery of antigens in vivo to CD91 induces enhanced activation of the adaptive immune system. It was further found that a subset of CD11c^+^ lineage-negative (lin^−^) DCs expresses the scavenger receptor CD91 and that these DCs are primarily responsible for the T cell activation that occurs following CD91-mediated antigen uptake [22]. Therefore, based on this evidence, we intend to test if chimeric CD40L/HIV VLPs conjugated to C1q delivered by sublingual administration will enhance the binding and delivery of VLPs across the epithelial lining in the sublingual mucosa, promote efficient interaction of antigen with DCs, promote DC activation in the oral submucosa, and elicit efficient mucosal and systemic immune responses against HIV.

## 2. Materials and Methods

### 2.1. Animals, Antibodies and Reagents

Female C57BL/6 mice from Jackson Laboratory at Baylor College of Medicine were used at 8 weeks of age. All mice were maintained under specific pathogen-free conditions in the animal facilities of Baylor College of Medicine and in accordance with the animal protocol approved by Institutional Animal Care and Use Committee (IACUC). Antibodies including fluorochrome-coupled antibodies to mouse CD3e, CD91, Integrin β7, CCR9 and to human CD54, CD86, and MHC class II (MHCII) were purchased from BD Biosciences (San Jose, CA, USA) and eBiosciences (San Diego, CA, USA). Anti-C1q antibody was purchased from Thermo Fisher Scientific (Waltham, MA, USA). ELISA, ELISPOT, and intracellular cytokine staining kits were purchased from BD Biosciences (San Jose, CA, USA). Protein and peptide pool of HIV-1 gag and env were obtained from NIH Reagents Program.

### 2.2. Mammalian VLP Production

Production of HIV VLPs is similar as described by Hammonds et al. [23]. Briefly, HIV-1 Gag/Env VLPs were produced from the HEK-293T cell line engineered to express HIV-1 gag/env genes under a tetracycline-inducible expression system (the cell line was a generous gift from Dr. Spearman at Emory University). Chimeric CD40L/HIV VLPs were produced by additional transfection of CD40L into the VLP producing cell lines. Both cell lines were maintained in DMEM medium containing 10% Tet system approved FBS, 4 mM L-glutamine, 100 units/mL penicillin, 100 µg/mL streptomycin, 100 µg/mL zeocin, and 5 µg/mL blasticidin. Production of VLPs was induced by adding 2 µg/mL of doxycycline. At 4 days upon induction, the medium containing VLPs was collected. The medium was centrifuged at 2500 rpm for 20 min to remove cell debris, then filtered through a 0.45 µm filter, and subjected to ultracentrifugation at 120,000× *g* for 2 h through 20% sucrose cushion. The resuspended pellets were again ultracentrifuged at 120,000× *g* for 1 h. The supernatant was carefully removed, and the remaining pellet containing the VLPs was resuspended in PBS and stored at 4 °C.

### 2.3. Characterization of Chimeric C1q/CD40L/HIV VLPs

C1q protein purchased from Sigma-Aldrich (St. Louis, MO, USA) was conjugated to CD40L/HIV VLP by using a Pierce Controlled Protein-Protein Crosslinking Kit (Thermo Fisher Scientific, Waltham, MA, USA). To characterize C1q conjugated CD40L/VLPs, immunogold staining was performed to stain C1q on VLP surface and an electron microscope (EM) was used to observe the conjugation of C1q on VLP surface. To further quantify the amount of C1q conjugated on VLP surface, first C1q was conjugated with Alexa Fluor 488 (AF488, Thermo Fisher Scientific, Waltham, MA, USA): 10 μL 1 mg/mL AF488 (1.56 × 10^−8^ mol in total) with 50 μL of 1.17 mg/mL C1q (1.43 × 10^−10^ mol in total). Extra free AF488 were then dialyzed out against PBS. The conjugation ratio was determined by fluorescent reading and compared to AF488 standard curve. Next, C1q-AF488 was conjugated to CD40L/HIV VLP, and the free C1q-AF488 was removed by washing and collecting the conjugated VLP pellet after ultracentrifugation. The conjugation ratio of C1q to CD40L/HIV VLP was indirectly calculated as following: 200 μL of 2.8 mg/mL VLP, which equals to 5.6 × 10^10^ VLP particles (1 mg of VLP product contains approximately 10^11^ VLP particles [24]), was used to conjugate with C1q-AF488. The conjugation ratio was determined by fluorescent reading and compared to the C1q-AF488 standard curve, which is equal to 1.8 × 10^13^ molecules of C1q on 5.6 × 10^10^ VLP particles. Therefore, there are about 321 molecules of C1q on each VLP surface. Three different batches of C1q/CD40L/HIV VLP were produced and the average conjugation of C1q on VLP surface is calculated to be about 324 C1q on each VLP surface.

### 2.4. Sublingual Immunization

**Figure 1:**
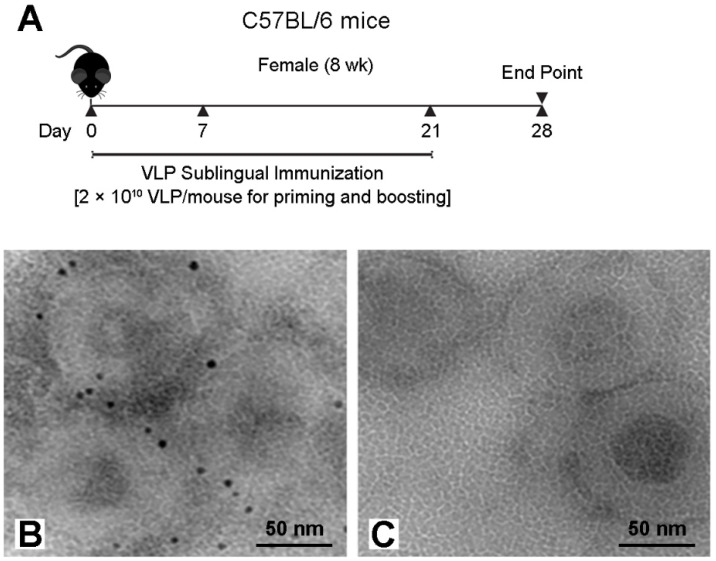
Sublingual immunization regimen with VLPs (**A**) and electron micrographs of immunogold labeling of C1q conjugation to CD40L/HIV VLPs. Rabbit anti-C1q antibody was diluted 1:100 and incubated with C1q conjugated CD40L/HIV VLPs (**B**), or CD40L/HIV VLPs (**C**). The secondary antibody was sheep anti-rabbit with gold conjugates diluted 1:100.

Sublingual immunization was done similarly with some modifications as described by Cuburu et al. [9]. C57BL/6 mice were randomly divided into three groups (*n* = 5/group) for CD40L/HIV VLPs, C1q/CD40L/HIV VLPs or PBS administration. Specifically, Mice were anesthetized and maintained with heads placed in ante flexion for the entire immunization procedure. 200 µg of VLPs (equal to approximately 2 × 10^10^ pseudovirions) were given to each mouse, with 5 µL applied to the sublingual mucosa every 20 min (total volume of 30 µL). Vaccines were administered on days 0, 7 and 21 (Figure 1A). Mice were sacrificed at day 28 and samples including sera, saliva, spleen, and cervical lymph node were harvested. All mice were maintained under specific pathogen-free conditions in the animal facilities of Baylor College of Medicine and in accordance with the animal protocol AN-3894 approved for this study by Institutional Animal Care and Use Committee (IACUC).

### 2.5. Determination of Dendritic Cell Activation

Human PBMC derived DCs were prepared and used to study DC activation by different VLP treatments as described before [25]. Selected DC activation markers CD54, CD86, and MHCⅡ were stained after stimulation with PBS (negative control), C1q (protein control, 5 µg/mL), CD40L/HIV VLPs (comparison control group, 5 µg/mL), or C1q/CD40L/HIV VLPs (experimental group, 5 µg/mL) for 24 h with day 5 GM-CSF and IL-4 differentiated DCs.

### 2.6. Enzyme Linked Immunosorbent Assay (ELISA)

Specific antibody levels in sera and saliva were determined by ELISA kit according to the manufacturer’s protocol (BD Biosciences, San Jose, CA, USA). To detect env and gag specific antibodies, microtiter plates were coated with either 3 µg/mL of env protein or gag protein per well (NIH AIDS Research and Reference Reagent Program). Protein coated plates were incubated overnight at 4 °C. After discarding coating solution, the plates were blocked in PBS containing 10% CS (calf serum) at RT for 1 h. Plates were then thoroughly washed with PBST. 2- or 10-fold serially diluted samples was added into each well and incubated at RT for 2 h. After incubation period, the plates were thoroughly washed again. The plates were then treated with goat anti-mouse IgA, or IgG-HRP conjugated antibodies for 1 h at RT. After all unbound conjugates were washed away, ABTS colorimetric substrate solution (Sigma-Aldrich, St. Louis, MO, USA) was added into each well. HRP enzyme reaction was stopped by the addition of a 1% SDS solution, and the OD values were read at 450 nm wavelength (against reference at 490 nm) in a microtiter reader (EL800, Bio-Tek Instruments, Winooski, VT, USA). The titers of detected samples were defined as the reciprocal of the highest sample dilution yielding an absorbance value at least equal to threefold that of background.

### 2.7. ELISPOT Assay

Plate-bound PVDF membranes (Millipore Corporation, Billerica, MA, USA) were pre-treated with 70% ethanol. Ethanol was removed and the membranes were washed with PBS. Anti-mouse IFN-γ coating antibody (BD Biosciences) was applied to the membranes and incubated overnight at 4 °C. The coating antibody was decanted, the plates were washed with PBST, and then blocked with PBS containing 10% CS for 1 h. Duplicates of 10^6^ splenocytes treated with 2 µM env or gag peptides pools (NIH Reagents) in 200 µL RPMI-1640 complete media were added to each well, and incubated at 37 °C overnight in a humidified 5% CO_2_ incubator. The cell suspensions were discarded and the plate was washed with distilled water and PBS to remove all splenocytes. Biotinylated anti-mouse IFN-γ detection antibody was added into each well and incubated for 2 h at RT. Plates were washed with PBST and incubated with avidin-HRP (BD Bioscience) for 15 min followed by five additional washes with PBST. Soluble HRP substrate, 3-amino-9-ethylcarbazole (AEC), in citrate buffer (Sigma) was added to each well and incubated at RT for 1 h. Spots were counted by ZellNet Consulting Inc (Fort Lee, NJ, USA).

### 2.8. Immunofluorescence Staining

Lymph nodes extracted from vaccinated mice were frozen in O.C.T media (Sakura). The samples were processed into 10 µm tissue slides using a cryostat cutter (Leica, Manheim, Germany). Tissue slides were fixed in cool acetone and stored at −80 °C until use. For immunostaining, tissue slides were rehydrated and incubated for 1 h in a blocking buffer containing PBS and 10% CS. Next, the slides were treated with fluorescent-labeled primary antibodies of anti-CD3e, anti-integrin β7, and anti-CCR9 antibodies for 2 h at RT in the blocking buffer. Following incubation, the slides were thoroughly washed with PBS-T, and then prepared for fluorescent detection. All images from the stained tissue slides were captured by using a Leica fluorescent microscope (Model: DM6000 B, Leica, Germany) equipped with a 100W xenon lamp and matched fluorescent filters.

### 2.9. Statistical Analysis

Data from treated and control groups were analyzed and results presented as the arithmetic mean ± standard error of mean (SEM). Statistical analyses were done with Student’s Unpaired T-test or One-way ANOVA, and Tukey Post-hoc test, for comparison of multiple groups. Kruskal-Wallis test or Mann-Whitney test was used for non-parametric data. Graphpad Prism was used to calculate statistics (Graphpad Software, Inc., La Jolla, CA, USA). A value of *p* < 0.05 was considered significant.

## 3. Results

### 3.1. Characterization of Chimeric C1q Conjugated CD40L/HIV VLPs

To characterize C1q conjugation to HIV Gag, Bal envelope gp160, CD40L VLPs (CD40L/HIV VLPs), we performed C1q specific immunogold staining on VLPs alone and C1q conjugated CD40L/HIV VLPs. As shown in Figure 1B, gold particles were detected on C1q conjugated CD40L/HIV VLPs, but not on unconjugated CD40L/HIV VLPs (Figure 1C). To quantify the amount of C1q conjugated to the CD40L/HIV VLP surface, we conjugated C1q to AF488 and determined the ratio of C1q to AF488 to be 1:6.7 (C1q to AF488 respectively), in reference to an AF488 standard curve. Based on this ratio, we conjugated AF488 conjugated C1q (AF488-C1q) to CD40L/HIV VLPs to calculate the indirect conjugation ratio of C1q to CD40L/HIV VLPs to be approximately 324:1 (C1q to CD40L/HIV VLPs respectively). This assay was repeated in triplicate with the C1q to CD40L/HIV VLPs ratio ranging from 300 to 350 and a mean ratio of 324 C1q proteins to 1 CD40L/HIV VLPs.

### 3.2. Characterization of Murine Sublingual Mucosal Lining and C1q Conjugated VLP Binding

**Figure 2:**
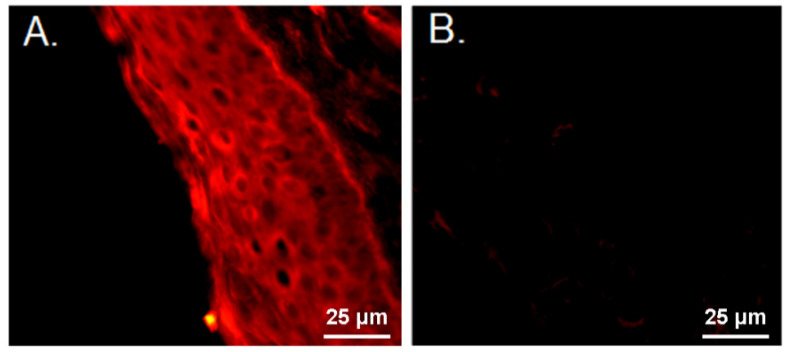
Immunofluorescent staining of CD91 expression on the sublingual mucosal lining. Transverse sections of sublingual paraffin embedded tissues were stained with (**A**) anti-CD91 antibody, or (**B**) isotype control antibody.

Receptors specific to C1q are required at or near the site of immunization so we initially stained mouse sublingual mucosa for the expression of CD91. By using PE conjugated anti-CD91, we found that CD91 is prevalent in the sublingual mucosal lining evidenced by immunofluorescence staining in transverse sections of sublingual tissue specimens from a C57BL/6 mouse (Figure 2A,B). After confirming the presence of a C1q receptor in the sublingual mucosa, we next sought to confirm VLP uptake by antigen presenting cells (APCs) after sublingual administration.

**Figure 3:**
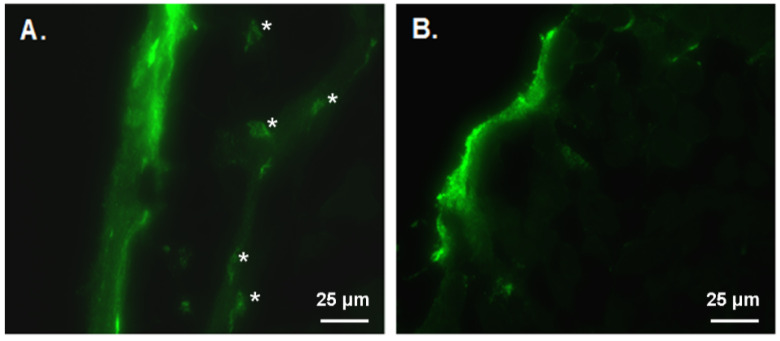
C1q conjugated CD40L/HIV VLPs preferentially enter the sublingual mucosal and bind to DC-like cells in the sub-mucosal. AF488 labeled CD40L/HIV VLPs were applied to sublingual surface and tissues were collected 120 min later. DC-like cells indicated by asterisks (*). (**A**) AF488 labeled C1q conjugated CD40L/HIV VLPs, (**B**) AF488 labeled CD40L/HIV VLPs.

To determine if C1q conjugated CD40L/HIV VLPs have the preferential ability to enter the sublingual surface layer. AF488 labeled C1q conjugated CD40L/HIV VLPs or CD40L/HIV VLPs were first applied to sublingual surface. After 120 min, sublingual tissues were collected and sections of sublingual tissues were observed under florescence microscope to determine whether any surface applied florescence labelled VLPs can enter to the sub-mucosal tissues and whether they preferentially bind to any of the cells in the sub-mucosal layer. We found that AF488 labeled C1q conjugated CD40L/HIV VLPs showed enhanced green florescence in the sub-mucosal layer and specifically in cells exhibiting dendritic cell like morphology (Figure 3A). Whereas, no green fluorescence in the sub-mucosal level was detected when AF488 labeled VLPs were administered without C1q conjugation (Figure 3B).

### 3.3. Sublingual Immunization with C1q Conjugated VLPs Induced Enhanced Mucosal Immune Responses and Antigen Specific Cellular Immune Responses

**Figure 4:**
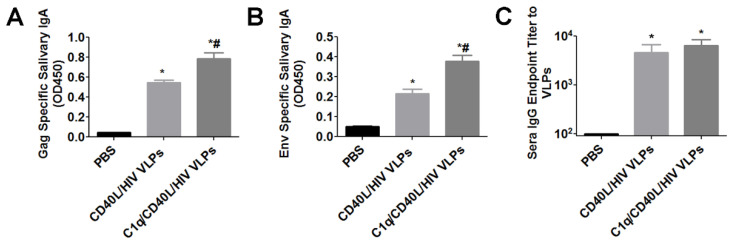
Determination of antigen specific IgA from salivary washes and sera IgG after oral sublingual immunization with chimeric C1q conjugated VLPs. ELISA plates were coated with purified HIV Pr55 Gag or HIV SF162 Env at 3 µg/mL. Salivary washes from mice at 4 weeks post immunization were diluted 1:4 to determine (**A**) HIV Pr55 Gag specific and (**B**) HIV Sf162 Env specific IgA. (**C**) Sera IgG endpoint titer to CD40L/HIV VLPs. ELISA plates were coated with purified CD40L/HIV VLPs at 3 µg/mL and subsequently incubated with diluted sera from the indicated groups. *n* = 5 mice for each group. Error bars represent the standard deviation. * indicates *p* < 0.05 by One-way ANOVA and Tukey Post Hoc Test compared to PBS. # indicates *p* < 0.05 by One-way ANOVA and Tukey Post Hoc Test compared to CD40L/HIV VLPs.

To determine whether C1q conjugated VLP sublingual immunization can induce enhanced mucosal immune responses, C57BL/6 mice were immunized with PBS, CD40L/HIV VLPs, or C1q conjugated CD40L/HIV VLPs. Mice were immunized via the sublingual route with 200 μg of VLPs at weeks 0, 1 and 3. At week 4, saliva was used to determine mucosal HIV antigen-specific IgA titers. Both VLPs alone and C1q conjugated CD40L/HIV VLPs induced significantly higher Gag and Env specific IgA compared to PBS (Figure 4A,B). In addition, C1q conjugated CD40L/HIV VLPs induced significantly higher Gag (0.788 vs. 0.546, *p* < 0.05) and Env (0.381 vs. 0.217, *p* < 0.05) specific IgA compared to VLPs alone. Interestingly, although CD40L/HIV VLPs and C1q/CD40L/HIV VLPs groups showed no significant difference, they induced significantly higher VLP specific IgG in sera compared to PBS group (Figure 4C).

**Figure 5:**
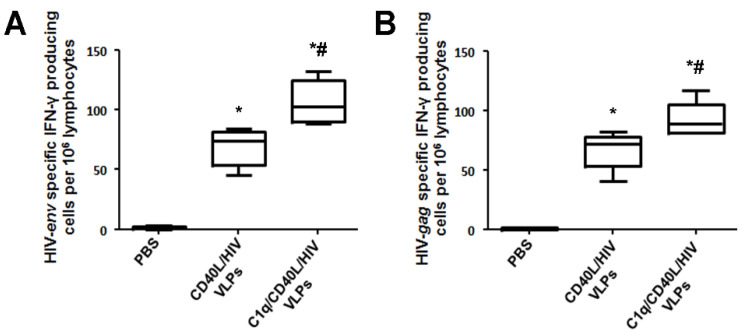
Induction of HIV env or gag specific splenocytes after sublingual inoculation with C1q conjugated CD40L/HIV VLPs. C57BL/6 mice (*n* = 5 mice per group) were immunized with PBS, CD40L/HIV VLPs, or C1q conjugated CD40L/HIV VLPs at day 0, and then boosted. One week from the last boost, splenocytes were prepared and amplified for 7 days with IL-2 and the indicated peptides. ELISPOT plates were coated with anti-IFN-γ coating antibody and splenocytes stimulated with either HIV gag or env peptides pools were added and incubated for 12 h. After thoroughly washing, a secondary antibody was incubated with AEC substrate; IFN-γ-secreting cells were manifested as red dots on the membrane, and counted by an image system. The numbers of (**A**) env specific, and (**B**) gag specific IFN-γ-secreting splenocytes were determined. Error bars represent the standard deviation. * indicates *p* < 0.05 by One-way ANOVA and Tukey Post Hoc Test compared to PBS. **#** indicates *p* < 0.05 by One-way ANOVA and Tukey Post Hoc Test compared to CD40L/HIV VLPs.

Splenocytes collected from the immunized mice were stimulated with HIV Gag or HIV Env consensus B peptide pools, and measured for IFN-γ by ELISPOT. As the number of HIV Gag or HIV Env specific splenocytes after sublingual immunization are limited, we used the Ag-specific T-cell enrichment method in the presence of IL-2 and HIV Gag or HIV Env specific peptides to amplify HIV Gag or HIV Env specific T cells for 7 days. Increased numbers of Env-specific IFN-γ producing cells were detected with CD40L/HIV VLP immunization compared to control. In addition, splenocytes from mice immunized with C1q conjugated CD40L/HIV VLPs had a greater number of IFN-γ specific spots compared to both PBS and CD40L/HIV VLPs alone (Figure 5A). A similar trend occurred with Gag peptide induced IFN-γ, in that a greater number of spots were detected from CD40L/HIV VLP immunized mice compared to PBS control after Gag stimulation (Figure 5B). Additionally, C1q conjugated CD40L/HIV VLPs had a further increase in detected spots compared to splenocytes from VLP only immunized mice (A, 102.08 vs. 73.44, *p* < 0.05; B, 89.06 vs. 71.88, *p* < 0.05).

### 3.4. C1q Conjugated VLPs Induced Enhanced Lymphocyte Homing to the Gut Mucosa

**Figure 6:**
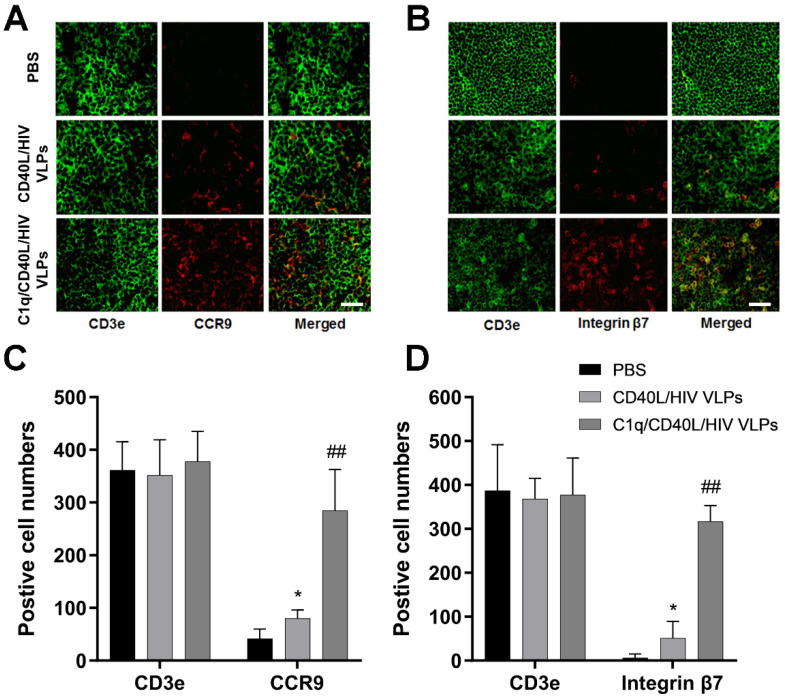
Determination of homing of mucosal T cells. Mice were inoculated with PBS, CD40L/HIV VLPs, and C1q conjugated CD40L/HIV VLPs at sublingual site for 7 days. Cervical lymph nodes were extracted and processed with standard frozen section protocol. The samples were stained with AF488 labeled anti-CD3e antibody and PE labeled anti-Integrin β7 or CCR9 antibodies. Lymphocytes from mouse cervical lymph node sections were co-stained with (**A**) CD3e and CCR9 antibodies or with (**B**) CD3e and integrin β7 antibodies. Scale bar, 25 μm. (**C**,**D**) The quantifications for immunofluorescence staining were determined by counting the positive cell numbers in each sample. Five random fields were selected and used to count numbers for each sample. Error bars represent means ± SEM. Data were analyzed by using Two-way ANOVA and unpaired Student’s *t* test. * indicates *p* < 0.05 compared to PBS. ## indicates *p* < 0.01 compared to CD40L/HIV VLPs.

In order to characterize the T cells involved in mucosal homing, mice were inoculated with PBS, CD40L/HIV VLPs, or C1q conjugated CD40L/HIV VLPs for 7 days; afterwards, cervical lymph nodes were extracted and processed for frozen sections. Tissue sections were stained with AF488 labeled anti-CD3e, and either PE labeled anti-integrin β7 or anti-CCR9 antibodies. The expression and co-expression of molecules of interest was visualized by fluorescence microscopy. Our results showed high expression of both integrin β7 and CCR9 on the surface of T cells in mice immunized with VLPs (Figure 6A,B). Although the CD3e positive cells did not show obvious differences, C1q/CD40L/HIV VLPs group suggested significant increases of CCR9 and integrin β7 expression compared to CD40L/HIV VLPs group (CCR9, 284 vs. 80, *p* < 0.01; integrin β7, 316 vs. 51, *p* < 0.01) (Figure 6C,D). As a negative control, PBS did not induce either CCR9 or Integrin β7 expression.

### 3.5. C1q Enhances VLP Activation of DCs

**Figure 7:**
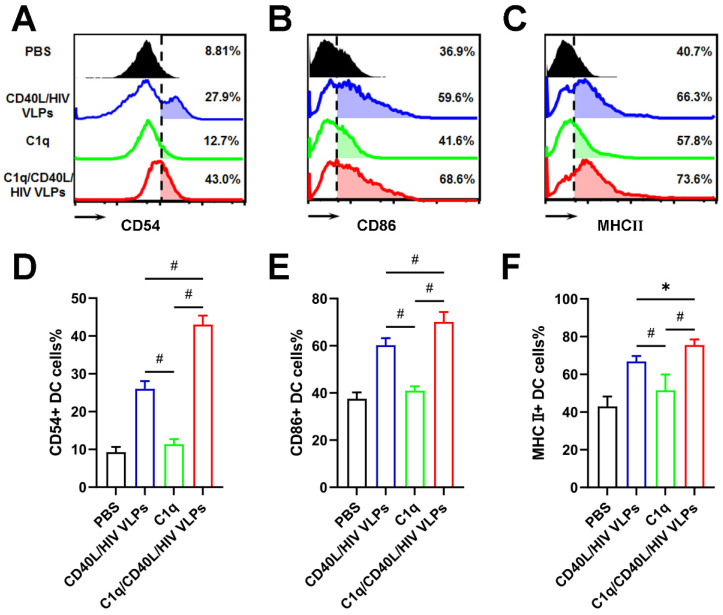
C1q conjugated VLPs enhances human DC activation markers. Human PBMC derived DCs were prepared and treated with 5 µg/mL CD40L/HIV VLPs, C1q conjugated CD40L/HIV VLPs (C1q/CD40L/HIV VLPs), C1q protein alone, or PBS for 24 h. After treatment, relative expression of plasma membrane DC activation markers (**A**) CD54, (**B**) CD86, and (**C**) MHCⅡ were measured by flow cytometry assay. (**D**–**F**) The quantifications for flow cytometry data of DC activation markers. Experiments were independently repeated three times. Error bars represent means ± SEM. Statistics analyses were performed by using One-way ANOVA and Tukey Post Hoc Test. *, *p* < 0.05; #, *p* < 0.01.

To verify that C1q adjuvant also affects human DCs, human PBMC derived DCs were treated with CD40L/HIV VLPs and analyzed by flow cytometry for activation markers CD54, CD86, and MHCⅡ (Figure 7A–C). By itself, C1q had a similar effect as PBS control on activation of the DCs. However, CD40L/HIV VLPs, both alone and C1q conjugated, efficiently activated human DCs. In addition, C1q conjugated CD40L/HIV VLPs had increased activation of all three markers when compared to unconjugated CD40L/HIV VLPs (CD54, 43.0% vs. 26.0%, *p* < 0.01; CD86, 70.1% vs. 60.2%, *p* < 0.01; MHCⅡ, 75.6% vs. 66.8%, *p* < 0.05;) (Figure 7D–F). These data indicate that C1q conjugated CD40L/HIV VLPs are capable of activating human DCs, making them an ideal adjuvant candidate.

## 4. Discussion

Immune response after mucosal administration leads to antigen-specific secretory IgA production at various distant mucosal sites [26]. Several mucosal administration strategies, such as oral administration and nasal administration, have been explored, however, oral immunization provides an immunity restricted to the digestive tract and the nasal administration faced several safety concerns [27]. A new area of research based on the sublingual delivery of vaccines emerged due to the variations in cell composition from one mucosa to another, and the expression of different combinations of pattern recognition receptor by tissue specific epithelial or innate cells [28]. The rich infiltration of antigen-presenting dendritic cells into the sublingual mucosa makes it an attractive antigen delivery route in immunization that targets the mucosal immune system and avoids needles [29]. Virus-like particles (VLPs) comprising the HIV gp41 protein, as well as antigens from other viruses, delivered via the sublingual route have shown in mice to be highly immunogenic and protective [6,30,31]. Given that CD91 is highly expressed in the salivary gland epithelial cells as well as on the surface of monocytes and dendritic cells (DCs) [21,32], we generated a novel HIV VLP system by adding the C1q component, CD91 binding ligand, to enhance the efficiency of sublingual delivery of VLP vaccination.

**Figure 8:**
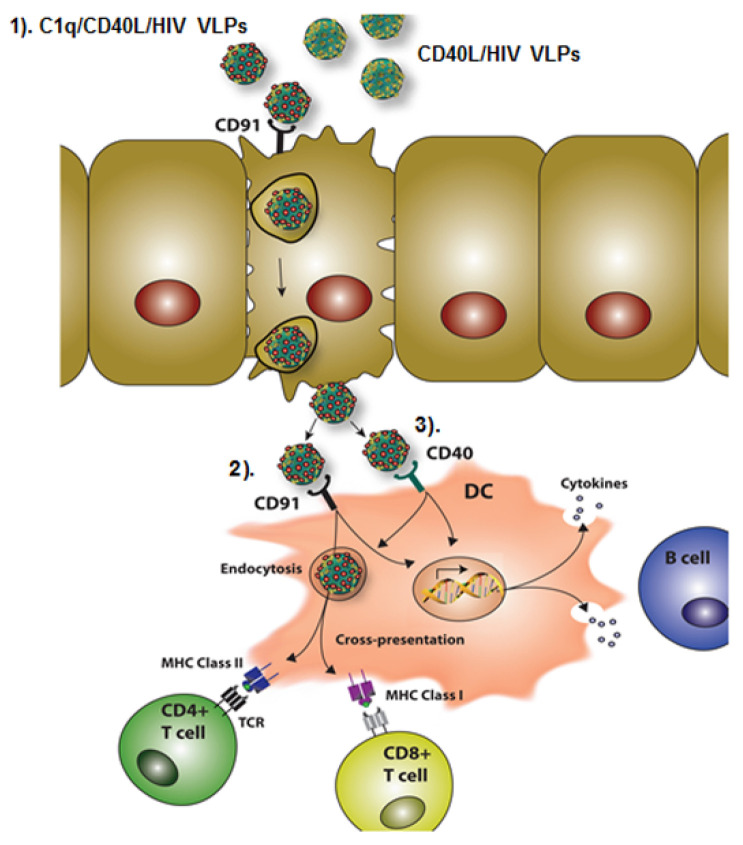
C1q conjugated CD40L/HIV VLPs (C1q/CD40L/HIV VLPs) induces mucosal immune response. (**1**) Preferential binding of C1q conjugated CD40L/HIV VLPs to epithelial layer CD91; (**2**) Preferential binding of C1q conjugated CD40L/HIV VLPs to the oral sub-mucosal DC surface receptor CD91; (**3**) Additional binding of CD40L to oral sub-mucosal DCs.

The aim of this study was to characterize C1q conjugated VLPs administered sublingually as a novel HIV vaccine. Our vaccine is composed of three parts: C1q, CD40L, and HIV Gag/Env VLPs, which exert different functions in inducing mucosal immune responses and as a whole, form a promising HIV vaccine. The proposed working model of our novel chimeric C1q/CD40L/HIV VLPs is illustrated in Figure 8. (1) C1q/CD40L/HIV VLPs preferentially target to CD91 expressed on the sublingual mucosal surface, which facilitates transport to the sub-mucosal layer; (2) novel C1q/CD40L/HIV VLPs preferentially target to CD91 expressed on a subset of DCs to facilitate endocytosis, and antigen cross-presentation or cytokine production enhancing the overall adaptive immune response; (3) C1q/CD40L/HIV VLPs not only activate CD91 expressing DCs but also activate CD40 receptor expressing DCs ensuring a broad spectrum of DC activation. The combined action of the multi-factorial APC activating components of our vaccine represents a novel sublingual immunogen which elicits both a humoral and cellular immune response to HIV.

The sub-mucosal layer in the sublingual mucosal has a dense network of dendritic-like cells with CD11c positive staining [33]. DCs are the most important APC in eliciting immune responses due to their highly efficient capture of antigen at the site of invasion, and subsequent migration to lymphoid organs for the optimal presentation of the invading pathogen components [34]. Therefore, a successful vaccine candidate should have the ability to efficiently activate DCs to ensure potent antigen presentation. Our previous study has shown that CD40L/SHIV VLPs have the ability to enhance DC activation by the interaction of CD40L with CD40 on DCs [19]. However, one of the obstacles in oral mucosal immunizations is antigen access to the sub-mucosal layer, especially for sublingual vaccination [35]. The murine sublingual mucosa consists of a keratinized pluristratified epithelium overlying a dense network of DCs; thus, special strategies should be performed to ensure maximal antigen uptake and minimal antigen tolerance [36,37]. In this study, we utilized a novel strategy by conjugated C1q proteins to our chimeric VLPs, taking advantage of the fact that CD91 is a C1q receptor and is able to promote the endocytosis of material coated with C1q. We expected that C1q coated CD40L/HIV VLPs as a mucosal antigen could facilitate the targeted delivery of antigen to the oral mucosal and oral sub-mucosal to enhance mucosal immune responses against HIV. Indeed, the facilitation induced by C1q was fully demonstrated by our results. An average of 324 C1q proteins are conjugated to the surface of one CD40L/HIV VLP, thus yielding a huge chimeric complex (C1q has a molecular weight of 410 kDa). Such a huge molecule is assumed to have a low ability to enter the sublingual surface; however, fluorescence microscopy showed this chimeric complex enters the sub-mucosal layer, where it was engulfed by DC-like cells. These data clearly indicate the role of C1q in facilitating transport of the whole complex through the mucosal layer and eventually binding to CD91-expressing DC-like cells, building a solid basis for our additional immunization studies.

Although C1q alone did not show much effect on activation of the markers such as CD54, CD86, and MHCⅡ when compared with PBS, C1q/CD40L/HIV VLPs showed a strong ability to activate these three markers, greater than that of CD40L/HIV VLPs alone, indicating the important role of C1q in our new chimeric CD40L/HIV VLP complex in activating DCs. These data confirmed our previous findings on CD40L as a potent DC activator and extended the use of CD40L in HIV vaccine design that CD40L can be used simultaneously with other functional molecules (e.g., C1q) to enhance the efficacy of vaccine immune responses [19]. Due to the constitutively high expression of CD40 and CD91 in dendritic cells [32,38,39,40], we didn’t measure the expression changes of CD40 or/and CD91 in DCs after treatment with C1q or VLPs, which could be a limitation of this study.

A potent acquired mucosal immune response requires the presence of large numbers of antibody secreting cells and activated T cells in mucosa [41]. Most activated T cells enter the mucosa upon their activation in lymph nodes and localize diffusely throughout mucosal lamina propria and the single layer epithelium. The above mentioned priming of mucosal T cells in mucosal tissues involves the expression of two molecules, α4β7-integrin and the chemokine receptor CCR9 to promote the specific homing [42]. Our studies demonstrated that C1q/CD40L/HIV VLPs induced expression of both CCR9 and Integrin β7, suggesting the effect of this novel complex on the priming of mucosal T cells, another reason for the potent immune responses elicited by C1q/CD40L/HIV VLPs.

Here we report for the first time that C1q can be conjugated to VLPs as a strategy to induce a robust humoral and cell mediated HIV Gag and Env specific response in the presence of CD40L. HIV vaccines administrated sublingually includes HIV-1 gp41 and adenoviral vector encoding HIV-1 Env or Gag; however, none of these induced both mucosal and cellular immune responses [6,7,8]. Interestingly, an HIV-1 Env vaccine was only reported to produce a humoral response, while a Gag vaccine elicited only cellular immune response [7,8]. Thus our study complements and extends on earlier work. The DCs underlying the mucosa rely on a number of specialized receptors to facilitate the uptake and intracellular accumulation of antigens. Studies have demonstrated that the targeted delivery of antigens in vivo to CD91 induces enhanced activation of the adaptive immune system [22]. Therefore, our novel chimeric C1q/CD40L/HIV VLPs targeted to CD91 on the sublingual mucosal and subsequently encounter sub-mucosal DCs, of which a subset express CD91. The unique strategy of this study is that the antigen components (HIV Env/Gag), the DC activator (CD40L), and antigen penetration facilitator (C1q) reside in the same particle in their natural state, stimulating more robust DC activation and thus more potent humoral and cellular immune responses than previously reported work.

Our novel HIV vaccine candidate induces a potent mucosal and cellular immune response after sublingual administration. Our data indicate the development of a mucosal targeting antigen with enhanced trans-epithelial properties to facilitate the contact of vaccine antigens with the sublingual epithelium and sub-mucosa by needle-free immunization is likely to become a major milestone for the future development of universal oral mucosal vaccines. The improved chimeric C1q conjugated CD40L/HIV VLPs warrant further study in their ability to induce protective immunity by viral challenge in the rhesus macaque model.

## Data Availability

Data is contained within the article.
